# Galectin-1 expression in the serum and placenta of pregnant women with fetal growth restriction and its significance

**DOI:** 10.1186/s12884-020-03477-8

**Published:** 2021-01-06

**Authors:** Xiao-Xiao Jin, Xiang Ying, Min-Yue Dong

**Affiliations:** 1 Department of Reproductive Genetics, Women’s Hospital of Zhejiang University, No. 1 of Xueshi Road, Shangcheng District, Hangzhou, 310006 China; 2 Department of Obstetrics, Taizhou Hospital, Zhejiang University, Taizhou, 317000 China

**Keywords:** Fetal growth restriction, Pregnant women, Galactose agglutination 1, Serum, The placenta

## Abstract

**Background:**

This study aims to investigate galectin-1 (Gal-1) expression in the serum and placenta of pregnant women with fetal growth restriction (FGR) and its significance.

**Methods:**

Thirty-one pregnant women with single-birth FGR but without comorbidities, eight pregnant women with FGR and preeclampsia (PE), and eight pregnant women with FGR and gestational diabetes mellitus (GDM) were enrolled as the study group, while 20 pregnant women with normal singleton pregnancy in the same period were enrolled as the control group. The serum Gal-1 level was detected using an enzyme-linked immunosorbent assay (ELISA), and Gal-1 expression in the placenta was detected by western blot.

**Results:**

The results revealed that, compared with the control group, the serum Gal-1 level decreased in the women with FGR without comorbidities, and the difference was statistically significant (*P* < 0.001). Compared with the control group, the difference in serum Gal-1 expression in the FGR-PE group was not statistically significant (*P* = 0.29). The peripheral serum Gal-1 level decreased in the FGR-GDM group compared with the control group, and the difference was statistically significant (*P* < 0.001). The serum Gal-1 level was positively correlated with birth weight (r^2^ = 0.172, *P* < 0.01). Compared with the control group, the Gal-1 expression level decreased in the placenta of the pregnant women with FGR without comorbidities (*P* < 0.05).

**Conclusions:**

Gal-1 exhibits low expression in the serum and placenta of pregnant women with FGR. In addition, Gal-1 may be involved in the pathogenesis of FGR and could represent a new diagnostic marker of the disease.

**Supplementary Information:**

The online version contains supplementary material available at 10.1186/s12884-020-03477-8.

## Background

Fetal growth restriction (FGR) refers to the failure of a fetus to reach its growth potential in the uterus due to various factors. It is a common complication during the perinatal period [1]. The pathogenesis of FGR is yet to be fully determined, though it is thought to correlate with various factors, such as maternal complications, age, nutritional deficiency, fetal chromosomal abnormalities, intrauterine infection, placental vascular endothelial damage, abnormal trophoblast differentiation, and inflammatory immune activation [2–4]. Placental factor is one of the main causes of FGR, and genes that regulate the functions of the placenta and trophoblasts, as well as changes in protein expression levels, are closely correlated with FGR [5].

Galectin is a family of soluble animal lectins that can specifically bind with the β-galactoside complex to regulate a variety of biological processes [6]. Galectin is involved in regulating maternal-fetal immune tolerance and promoting angiogenesis in placenta formation; as such, it plays an important role in placenta formation and pregnancy maintenance [7]. Galectin-1 (Gal-1) is a member of the galectin family that is highly expressed in the placenta. Previous studies have shown that Gal-1 participates in the regulation of the biological behavior of trophoblasts and angiogenesis and in the establishment of the microenvironment of maternal-fetal immune tolerance at the maternal-fetal interface [8, 9]. Abnormal levels of Gal-1 are closely correlated with obstetric complications, such as premature delivery and preeclampsia [10, 11]. However, few studies have examined the role of Gal-1 in FGR.

Therefore, in the present study, Gal-1 expression in the serum and placenta of pregnant women with FGR was detected, and the correlation between the Gal-1 level and birth weight was investigated to yield further insight into its role in the pathogenesis of intrauterine FGR.

## Methods

### Study subjects

In the present study, 31 pregnant women with single-birth FGR but without comorbidities, eight pregnant women with FGR and preeclampsia (PE), and eight pregnant women with FGR and gestational diabetes mellitus (GDM) admitted to the Taizhou Hospital of Zhejiang Province from January 2017 to February 2019 were enrolled as the study group. Twenty pregnant women with normal singleton pregnancy without complications or comorbidities in the same period were enrolled as the control group. Diagnosis was made based on the following criterion: small for gestational age (SGA) infants (i.e., the birth weight of the fetus was less than the 10th percentile of the same gestational age). The fetal growth and development of the two groups were monitored by B-ultrasound.

In the present study, all programs were conducted according to International Ultrasound Society of Obstetrics and Gynecology (ISUOG) guidelines. The research plan was approved by the Ethics Committee of Taizhou Hospital of Zhejiang Province, and all participants provided signed informed consent.

### Inclusion and exclusion criteria

Inclusion criteria: (1) pregnant women diagnosed with single-birth FGR, (2) pregnant women > 18 years old, and (3) informed consent provided by the pregnant women. Exclusion criteria: (1) pregnant women with other pregnancy comorbidities and complications, (2) pregnant women with a history of smoking and drinking, (3) pregnant women with a history of assisted reproduction, and (4) pregnant women with incomplete medical information.

### Specimen collection

#### Peripheral blood samples

Prior to delivery, 4 ml maternal peripheral venous blood was drawn and centrifuged at 3500 rpm for 5 min at 4 °C. The serum was subsequently separated and placed in a refrigerator at − 80 °C for future use.

#### Placental tissues

The placental tissues were treated within 10 min of delivery. A full-thickness piece of placental tissue of 1.0 × 1.0 × 0.2 cm in size was cut from the attached umbilical cord and washed with normal saline three times, and the water was subsequently dried out using a gauze. The tissue was then placed in a refrigerator at − 80 °C for future use.

### Experimental reagents

Gal-1 antibodies were purchased from Abcam (U.K.). Goat anti rabbit secondary antibodies were purchased from Cell Signaling Technology (U.S.), and the Gal-1 ELISA kit was purchased from Santa Cruz Bicycles (U.S.).

### Sample test

#### Detection of serum Gal-1 level by enzyme-linked immunosorbent assay (ELISA)

The level of serum Gal-1 was detected according to the manufacturer of the ELISA kit’s instructions. The specific steps were as follows: (1) the standard of Gal-1 was diluted to the corresponding concentration series; (2) the samples were added; (3) the samples were incubated for 30 min at 37 °C, washed five times, and dried by shaking; (4) horseradish peroxidase (HRP) was added; (5) step 3 was repeated; (6) the chromogenic agent was added, and the samples were places in the dark and incubated for 15 min at 37 °C before a termination solution was added; (7) the optical density (OD) value of each well at 450 nm was detected, and a “concentration-OD value” curve was drawn to calculate the concentration of the sample.

#### Detection of Gal-1 expression in the placenta by western blot

Placental protein was extracted by radio-immunoprecipitation assay (RIPA), and 1 ml tissue lysate and 10 μl phenylmethylsulfonyl fluoride (PMSF) were added to each 100 mg placenta tissue. The tissues were subsequently homogenized on ice and lysed for 5 min three times. The protein concentration was determined using bicinchoninic acid (BCA). The proteins to be tested were separated by sodium dodecyl sulfate polyacrylamide gel electrophoresis (SDS-PAGE) and transferred to a PVDF membrane, followed by 1 h blocking at room temperature (RT) in 7% non-fat milk of PBS with 0.07% Tween-20. Rabbit polyclonal antibody Gal-1 (Abcam, ab25138) was added to the blots and incubated overnight at 4 °C. Following this, the membrane was washed with tris-buffered saline (TBS) and Tween 20, goat anti-rabbit secondary antibody (Abcam, ab205718) was added, and the membrane was placed in room temperature conditions and incubated for 2 h. The membrane was then washed with TBS and exposed with enhanced chemiluminescence (ECL). GAPDH was used as an internal loading control. The band intensity was quantified using Image J software.

### Statistical analysis

Data were analyzed using statistical software SPSS 20.0. Data were expressed as mean ± standard deviation (x ± SD). Count data were expressed as a percentage (%). The test of normality was conducted with a W-test. The homogeneity of variance was tested using an F-test. A multi-group comparison was conducted using univariate analysis of variance, and back testing was conducted using the least significant difference (LSD). The non-normally distributed means of multiple samples or normally distributed means of multiple samples with heterogeneity of variance were compared using a nonparametric test. Count data were compared using a Chi-square test. The correlation analysis was conducted using a Pearson’s correlation analysis. *P* < 0.05 was considered statistically significant.

## Results

### General situations

In the present study, 31 pregnant women with single-birth FGR without comorbidities, eight pregnant women with preeclampsia (PE) and FGR, and eight pregnant women with FGR and gestational diabetes mellitus (GDM) admitted to the Taizhou hospital, Zhejiang Province from January 2017 to February 2019 were enrolled as the study group. Twenty pregnant women with normal singleton pregnancy without complications or comorbidities in the same period were enrolled as the control group.

The age, height, body weight, gestational age, and birth weight of all the patients with FGR and normal pregnant women were determined, and the results are shown in Table 1. Compared with the control group, the differences in age, height, and body weight of three FGR groups were not statistically significant (*P* > 0.05 for all). The gestational ages were 37.16 ± 0.86, 37.50 ± 0.77, and 35.23 ± 1.18 weeks in FGR without comorbidities group, FGR-GDM, and FGR-PE group, respectively, and 38.90 ± 1.14 weeks in the control group. The in gestational age between the control group and all three FGR groups were statistically significant (*P* < 0.05). The birth weights were 2290.81 ± 221.39, 2406.88 ± 64.42, and 1850.00 ± 241.07 g in the FGR without comorbidities group, FGR-GDM group, and FGR-PE group, respectively, and 3303.00 ± 307.38 g in the control group. The differences in gestational age between the control group and the three FGR groups were statistically significant (*P* < 0.05).

**Table 1:** The general information in each group

Index	Control group	FGR without comorbidities	FGR-GDM	FGR-PE	P1	P2	P3
n	20	31	8	8	-	-	-
Age (years)	27.65±4.36	26.74±5.08	28.23±5.30	29.75±5.42	0.51	0.67	0.29
Height (cm)	159.10±5.29	157.78±5.96	157.25±3.49	158.38±4.03	0.42	0.37	0.73
Weight (kg)	68.40±9.84	63.98±8.22	64.58±10.59	71.16±9.93	0.09	0.37	0.51
Gestational age (Weeks)	38.90±1.14	37.16±0.86	37.50±0.77	35.23±1.18	<0.001	0.01	<0.001
Birth weight (g)	3303.00±307.38	2290.81±221.39	2406.88±64.42	1850.00±241.07	<0.001	<0.001	<0.001

Note:

P1: Control group vs. FGR without comorbidities group

P2: Control group vs. FGR-GDM group

P3: FGR-GDM vs. FGR-PE group

### Gal-1 expression in peripheral blood in the different groups

**Fig. 1:**
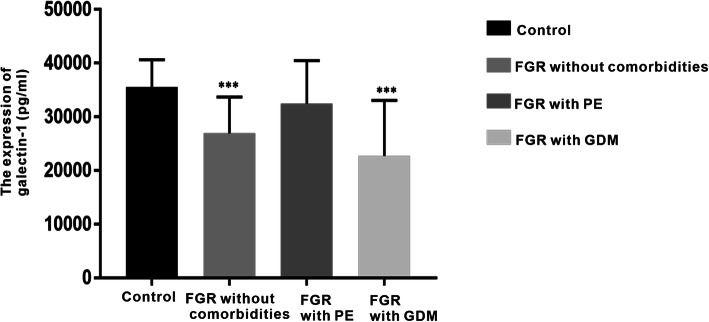
The expression of galectin-1 protein in peripheral blood in the different groups. The result showed that Gal-1levels were significantly decreased in FGR group and FGR with GDM group. But for FGR with PE group, the Gal-1 level was not significantly decreased.***: *P* < 0.001 when compared with control group

As shown in Fig. 1, the expression level of serum Gal-1 was 35,558.81 ± 5097.42 pg/ml in the control group and 26,951.35 ± 6751.42 pg/ml in the pregnant women with FGR without complications group, and the difference was statistically significant (*P* < 0.001). Compared with the control group (35,558.81 ± 5097.42 pg/ml), the peripheral serum Gal-1 level was 22,748.50 ± 10,318.48 pg/ml in the FGR-GDM group, and the difference was statistically significant (*P* < 0.001). However, the peripheral serum Gal-1 level was 32,447.25 ± 8043.88 pg/ml in the FGR-PE group, though the difference was not statistically significant when compared with the control group (35,558.81 ± 5097.42 pg/ml, *P* = 0.29). The results showed that the peripheral serum Gal-1 level decreased significantly in the FGR and FGR-GDM groups compared with the control group.

### The correlation between Gal-1 level and birth weight

**Fig. 2:**
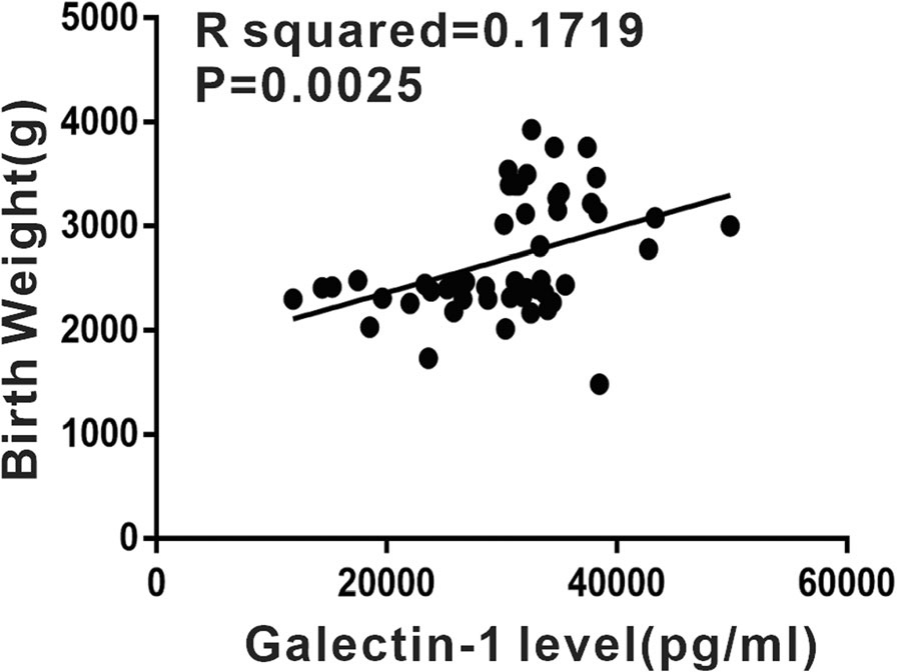
The correlation between galectin-1 protein level in peripheral blood and birth weight. The results revealed that serum galectin-1 level was positively correlated with birth weight

To further investigate the correlation between the Gal-1 level and birth weight, a Pearson’s analysis was conducted. The results revealed that the serum Gal-1 level was positively correlated with birth weight (r^2^ = 0.172, *P* < 0.01; Fig. 2).

### The expression of Gal-1 in the placenta

**Fig. 3:**
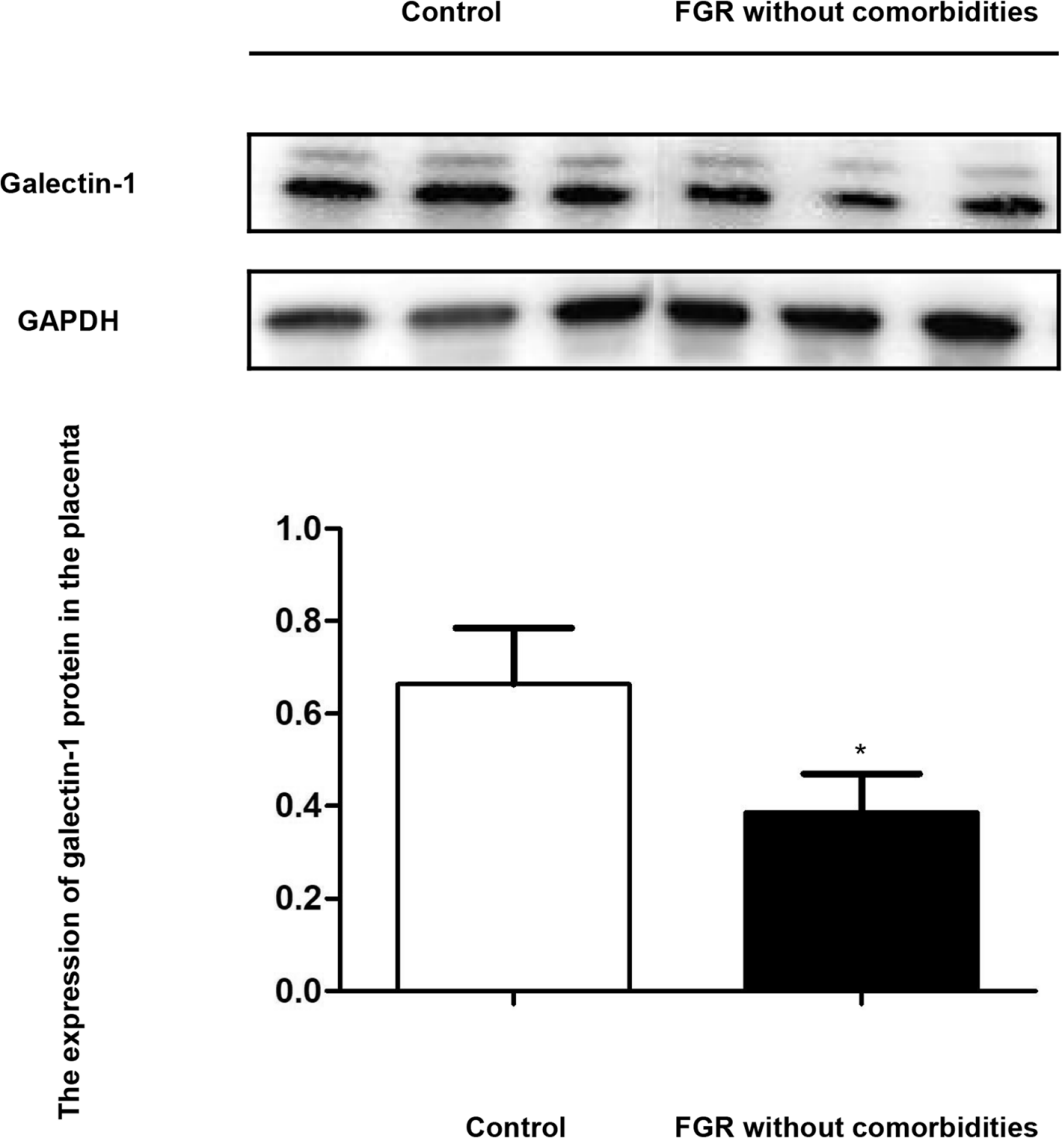
The expression of galectin-1 protein in the placenta detected by western blot. The results revealed that compared with the control group, the expression of galectin-1 in placental tissue decreased in the FGR group. *: *P* < 0.05 when compared with control group

The expression of Gal-1 in the placenta in the control group and FGR group was detected by western blot. The results revealed that the expression of Gal-1 in placental tissue decreased in the FGR group compared with the control group, and the difference was statistically significant (*P* < 0.05, Fig. 3, Additional files 1, 2, 3 and 4).

## Discussion

The results showed that the peripheral serum Gal-1 level decreased in the FGR group compared with the control group, and the difference was statistically significant. Furthermore, the serum Gal-1 level was found to be positively correlated with birth weight. The Gal-1 expression level in the placenta also significantly decreased in the FGR group compared with the control group.

The incidence and perinatal fatality rate of pregnant women with FGR has been steadily increasing in recent years. FGR can cause fetal distress, stillbirth, and other adverse perinatal outcomes and is correlated with long-term adult diseases, such as cardiovascular disease and metabolic disorder syndrome after birth. At present, there are a lack of diagnostic markers and specific treatments for the disease; therefore, in-depth research into FGR is necessary.

Gal-1 is encoded by the LGALS1 gene, which belongs to the soluble animal lectin family. The gene is located on human chromosome 22q13.1. Gal-1 is mainly found in the cytoplasm and is secreted to the outside of cells by non-classical pathways. It is involved in a number of pathological and physiological processes, including cell proliferation and apoptosis, cell adhesion, and angiogenesis. Gal-1 is expressed in all types of trophoblasts during pregnancy, and the expression of Gal-1 in the peripheral blood of pregnant women increases from the early stage of pregnancy, peaking in the middle and late stages of pregnancy [12]. This peak level is maintained until the third trimester [12]. In one study, it was found that the difference in Gal-1 expression in the placenta between the FGR group and the control group was not statistically significant [13]. However, the present study found that Gal-1 expression decreased in the placenta of pregnant women with FGR and other comorbidities. In addition, the expression of Gal-1 in the placenta and peripheral blood has been shown to increase in PE patients [14]. The present study found that the difference in the expression of Gal-1 in peripheral blood between the FGR-PE group and control group was not statistically significant. In light of this, it is thought that the serum Gal-1 level is high in pregnant women with PE, but does not significantly decrease in pregnant women with FGR-PE. In addition, it is thought that the serum Gal-1 expression of FGR in pregnancy is low. In one study, it was found that the expression of Gal-1 in the peripheral blood was increased in GDM patients [15]. Furthermore, Gal-1 is known to be highly expressed in diabetic mouse kidney and human retina tissues [16, 17]. This suggests that the high expression of Gal-1 in the peripheral blood of patients with GDM may be correlated with diabetes. The present study revealed that the serum Gal-1 expression in peripheral blood was low in patients with FGR in pregnancy. Therefore, serum Gal-1 expression may be correlated with FGR, which could, therefore, represent a new diagnostic marker.

The etiology of FGR is complex. At present, it is thought that the onset of FGR is correlated with inadequate uterine placental perfusion and fetal nutrition restriction. Between 30 and 40% of FGR etiologies remain unknown [18]. The formation and normal function of blood vessels at the maternal-fetal interface are highly important in the establishment and maintenance of pregnancy. Although the specific etiology and mechanism of FGR remain unclear, it has been argued that placental dysfunction is one of the main causes of FGR. One study noted that Gal-1 expression was abundant in the genital tract, particularly in the endometrium, decidua, and placenta, which suggests that Gal-1 plays an important role in pregnancy [19]. Gal-1 is a marker of vascular endothelial cell activation, and decreasing the expression of Gal-1 inhibits the biological behavior of endothelial cells [20, 21]. Freitag et al. observed that Gal-1 promoted the activation of decidual vessels through angiopoietin and other angiogenic factors, which altered the outcome of the embryo’s cessation of development [22]. In addition, Gal-1 has been found to be involved in the regulation of immune response, cell adhesion, invasion, and other pregnancy processes [23]. The expression of Gal-1 in the placenta was detected by western blot. The results of the present study revealed that the expression level of Gal-1 in the placenta decreased in patients with FGR. This suggests that Gal-1 may affect the erosive ability of the uterine spiral artery, cause poor placental vascular reconstruction, and decrease the placental exchange area and villus space, leading to placental hypoperfusion. Hence, it can be said that Gal-1 participates in the regulation of maternal and fetal immunity, and the biological behavior of trophoblasts and other pregnancy processes, subsequently leading to FGR.

The present study had the following limitations. First, this study was a retrospective study, not a randomized controlled trial; in addition, the study was not blinded. Therefore, there is a certain risk of bias. Second, the study was a single-center clinical trial, and the included sample size was small. Therefore, multi-center clinical trials with larger sample sizes are needed. Third, this study used both term and preterm placentas; however, placental phenotypes and gene expression may differ between term and preterm placentas because a number of factors may affect placental gene expression. Finally, in the present study, the serum level of Gal-1 in pregnant women at different gestational ages was not investigated. Therefore, further investigation in this respect is needed.

## Conclusion

Gal-1 exhibits low expression in the serum and placenta of pregnant women with FGR. Gal-1 may be involved in the pathogenesis of FGR and could represent a new diagnostic marker of the disease.

## Supplementary Information

**Additional file 1.** The expression of Glaectin-1 in control group (*n*=3).

**Additional file 2.** The expression of Glaectin-1 in FGR group (*n*=3 per group).

**Additional file 3.** A western blot analysis of GAPDH expression in control patients (*n*=3 per group).

**Additional file 4.** A western blot analysis of GAPDH expression in FGR group (*n*=3).

## Data Availability

The datasets used and/or analysed during the current study are available from the corresponding author on reasonable request.

## Abbreviations

Gal-1: Galectin-1
FGR: Fetal growth restriction
PE: Preeclampsia
GDM: Gestational diabetes mellitus
ELISA: Enzyme-linked immunosorbent assay
SGA: Small for gestational age
ISUOG: International Ultrasound Society of Obstetrics and Gynecology
HRP: Horseradish peroxidase
OD: Optical density
RIPA: Radio-immunoprecipitation assay
PMSF: Phenylmethylsulfonyl fluoride
BCA: Bicinchoninic acid
SDS-PAGE: Sodium dodecyl sulfate polyacrylamide gel electrophoresis
TBST: Tris-Buffered Saline and Tween 20
TBS: Tris buffered saline
ECL: Enhanced chemiluminescence
LSD: Least significant difference
