# Supplementary figures and images for: Galectin-1 expression in the serum and placenta of pregnant women with fetal growth restriction and its significance
Source: BMC Pregnancy Childbirth. 2021 Jan 6;21:14. doi: 10.1186/s12884-020-03477-8 (PMC7789211; doi:10.1186/s12884-020-03477-8)

**Additional file 1**


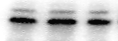


The expression of Glaectin-1 in control group (n=3).

Supplement: Supplementary file 1 — Additional file 1. The expression of Glaectin-1 in control group (n=3). [file 12884_2020_3477_MOESM1_ESM.docx]

**Additional file 2**


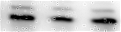


The expression of Glaectin-1 in FGR group (n=3 per group).

Supplement: Supplementary file 2 — Additional file 2. The expression of Glaectin-1 in FGR group (n=3 per group). [file 12884_2020_3477_MOESM2_ESM.docx]

**Additional file 3**


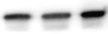


A western blot analysis of GAPDH expression in control patients (n=3 per group).

Supplement: Supplementary file 3 — Additional file 3. A western blot analysis of GAPDH expression in control patients (n=3 per group). [file 12884_2020_3477_MOESM3_ESM.docx]

**Additional file 4**


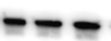


A western blot analysis of GAPDH expression in FGR group (n=3).

Supplement: Supplementary file 4 — Additional file 4. A western blot analysis of GAPDH expression in FGR group (n=3). [file 12884_2020_3477_MOESM4_ESM.docx]
